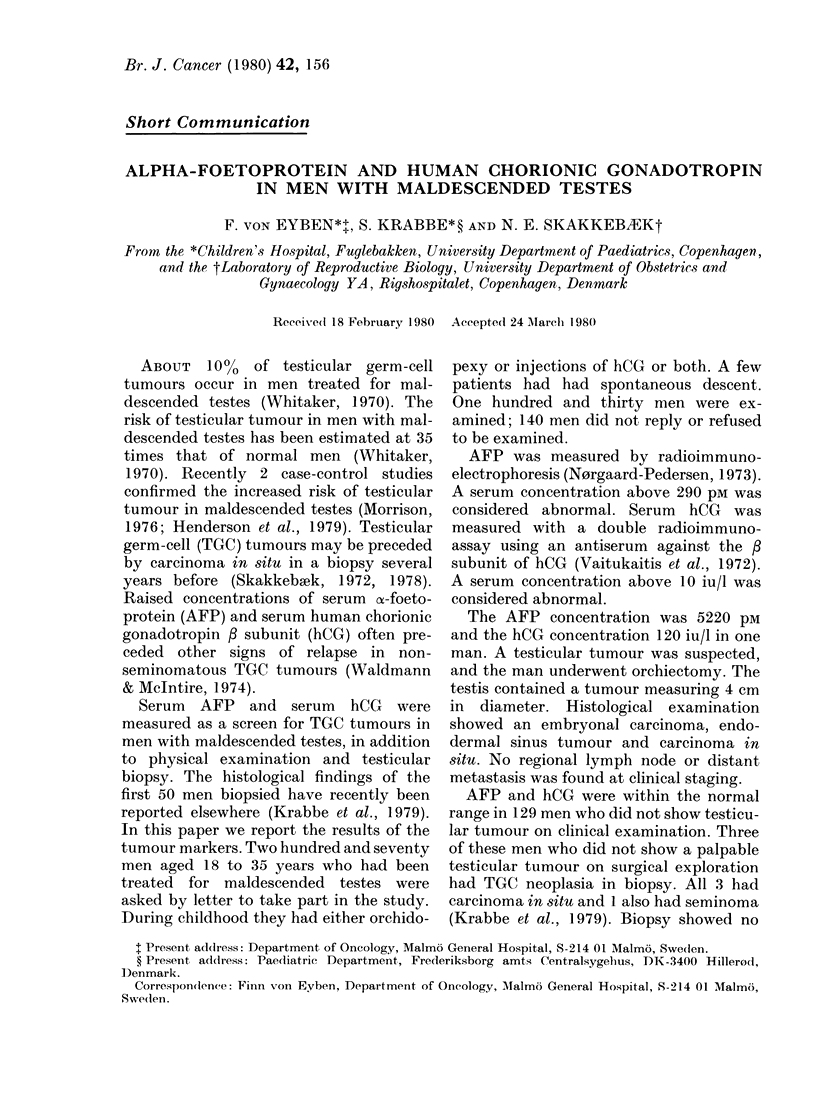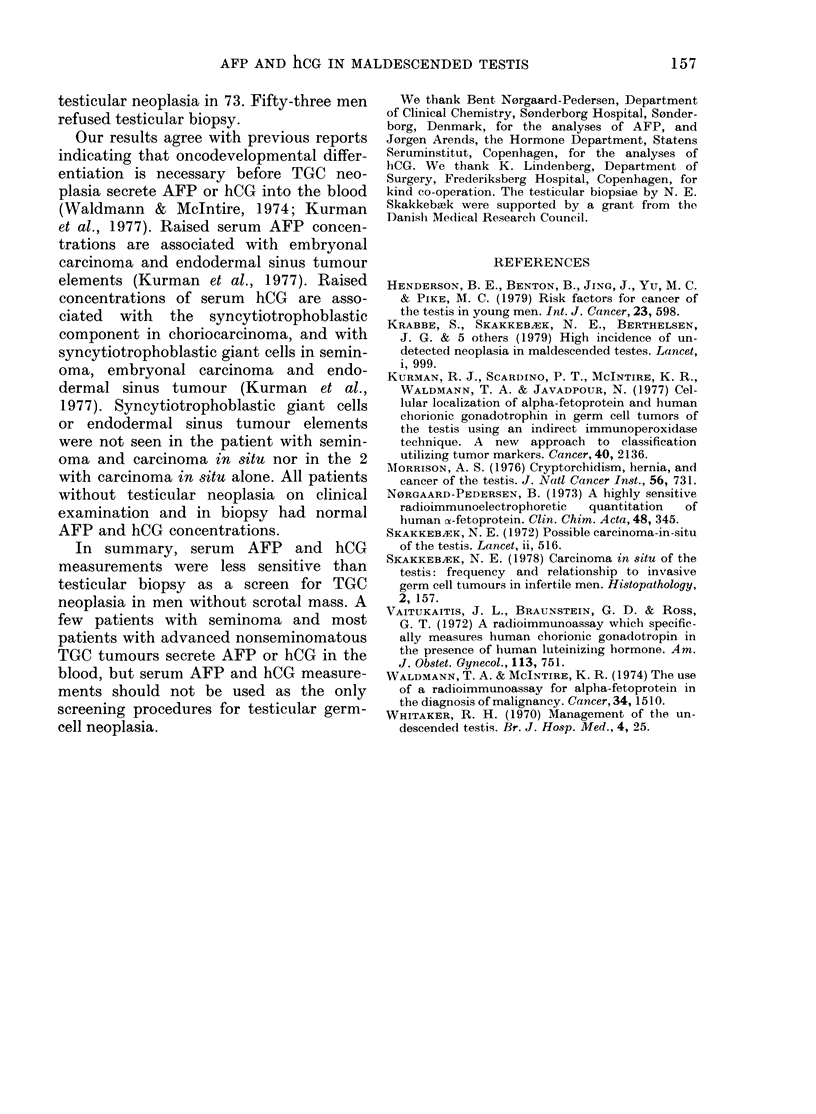# Alpha-foetoprotein and human chorionic gonadotropin in men with maldescended testes.

**DOI:** 10.1038/bjc.1980.214

**Published:** 1980-07

**Authors:** F. von Eyben, S. Krabbe, N. E. Skakkebaek


*Br. J. Cancer* (1980) **42**, 156

***Short Communication***

### ALPHA-FOETOPROTEIN AND HUMAN CHORIONIC GONADOTROPIN IN MEN WITH MALDESCENDED TESTES

F. VON EYBEN*‡, S. KRABBE*§ AND N. E. SKAKKEBÆK†

*From the *Children's Hospital, Fuglebakken, University Department of Paediatrics, Copenhagen, and the †Laboratory of Reproductive Biology, University Department of Obstetrics and Gynaecology YA, Rigshospitalet, Copenhagen, Denmark*

Received 18 February 1980 Accepted 24 March 1980

ABOUT 10% of testicular germ-cell tumours occur in men treated for maldescended testes (Whitaker, 1970). The risk of testicular tumour in men with maldescended testes has been estimated at 35 times that of normal men (Whitaker, 1970). Recently 2 case-control studies confirmed the increased risk of testicular tumour in maldescended testes (Morrison, 1976; Henderson *et al.*, 1979). Testicular germ-cell (TGC) tumours may be preceded by carcinoma *in situ* in a biopsy several years before (Skakkebæk, 1972, 1978). Raised concentrations of serum $\alpha$-foetoprotein (AFP) and serum human chorionic gonadotropin $\beta$ subunit (hCG) often preceded other signs of relapse in non-seminomatous TGC tumours (Waldmann & McIntire, 1974).

Serum AFP and serum hCG were measured as a screen for TGC tumours in men with maldescended testes, in addition to physical examination and testicular biopsy. The histological findings of the first 50 men biopsied have recently been reported elsewhere (Krabbe *et al.*, 1979). In this paper we report the results of the tumour markers. Two hundred and seventy men aged 18 to 35 years who had been treated for maldescended testes were asked by letter to take part in the study. During childhood they had either orchidopexy or injections of hCG or both. A few patients had had spontaneous descent. One hundred and thirty men were examined; 140 men did not reply or refused to be examined.

AFP was measured by radioimmunoelectrophoresis (Nørgaard-Pedersen, 1973). A serum concentration above 290 pM was considered abnormal. Serum hCG was measured with a double radioimmunoassay using an antiserum against the $\beta$ subunit of hCG (Vaitukaitis *et al.*, 1972). A serum concentration above 10 iu/l was considered abnormal.

The AFP concentration was 5220 pM and the hCG concentration 120 iu/l in one man. A testicular tumour was suspected, and the man underwent orchiectomy. The testis contained a tumour measuring 4 cm in diameter. Histological examination showed an embryonal carcinoma, endodermal sinus tumour and carcinoma *in situ*. No regional lymph node or distant metastasis was found at clinical staging.

AFP and hCG were within the normal range in 129 men who did not show testicular tumour on clinical examination. Three of these men who did not show a palpable testicular tumour on surgical exploration had TGC neoplasia in biopsy. All 3 had carcinoma *in situ* and 1 also had seminoma (Krabbe *et al.*, 1979). Biopsy showed no

‡ Present address: Department of Oncology, Malmö General Hospital, S-214 01 Malmö, Sweden.
§ Present address: Paediatric Department, Frederiksborg amts Centralsygehus, DK-3400 Hillerød, Denmark.
Correspondence: Finn von Eyben, Department of Oncology, Malmö General Hospital, S-214 01 Malmö, Sweden.

testicular neoplasia in 73. Fifty-three men refused testicular biopsy.

Our results agree with previous reports indicating that oncodevelopmental differentiation is necessary before TGC neoplasia secrete AFP or hCG into the blood (Waldmann & McIntire, 1974; Kurman *et al.*, 1977). Raised serum AFP concentrations are associated with embryonal carcinoma and endodermal sinus tumour elements (Kurman *et al.*, 1977). Raised concentrations of serum hCG are associated with the syncytiotrophoblastic component in choriocarcinoma, and with syncytiotrophoblastic giant cells in seminoma, embryonal carcinoma and endodermal sinus tumour (Kurman *et al.*, 1977). Syncytiotrophoblastic giant cells or endodermal sinus tumour elements were not seen in the patient with seminoma and carcinoma *in situ* nor in the 2 with carcinoma *in situ* alone. All patients without testicular neoplasia on clinical examination and in biopsy had normal AFP and hCG concentrations.

In summary, serum AFP and hCG measurements were less sensitive than testicular biopsy as a screen for TGC neoplasia in men without scrotal mass. A few patients with seminoma and most patients with advanced nonseminomatous TGC tumours secrete AFP or hCG in the blood, but serum AFP and hCG measurements should not be used as the only screening procedures for testicular germ-cell neoplasia.

We thank Bent Nørgaard-Pedersen, Department of Clinical Chemistry, Sønderborg Hospital, Sønderborg, Denmark, for the analyses of AFP, and Jørgen Arends, the Hormone Department, Statens Seruminstitut, Copenhagen, for the analyses of hCG. We thank K. Lindenberg, Department of Surgery, Frederiksberg Hospital, Copenhagen, for kind co-operation. The testicular biopsiae by N. E. Skakkebæk were supported by a grant from the Danish Medical Research Council.

### REFERENCES

HENDERSON, B. E., BENTON, B., JING, J., YU, M. C. & PIKE, M. C. (1979) Risk factors for cancer of the testis in young men. *Int. J. Cancer*, **23**, 598.

KRABBE, S., SKAKKEBÆK, N. E., BERTHELSEN, J. G. & 5 others (1979) High incidence of undetected neoplasia in maldescended testes. *Lancet*, i, 999.

KURMAN, R. J., SCARDINO, P. T., MCINTIRE, K. R., WALDMANN, T. A. & JAVADPOUR, N. (1977) Cellular localization of alpha-fetoprotein and human chorionic gonadotrophin in germ cell tumors of the testis using an indirect immunoperoxidase technique. A new approach to classification utilizing tumor markers. *Cancer*, **40**, 2136.

MORRISON, A. S. (1976) Cryptorchidism, hernia, and cancer of the testis. *J. Natl Cancer Inst.*, **56**, 731.

NØRGAARD-PEDERSEN, B. (1973) A highly sensitive radioimmunoelectrophoretic quantitation of human $\alpha$-fetoprotein. *Clin. Chim. Acta*, **48**, 345.

SKAKKEBÆK, N. E. (1972) Possible carcinoma-in-situ of the testis. *Lancet*, ii, 516.

SKAKKEBÆK, N. E. (1978) Carcinoma *in situ* of the testis: frequency and relationship to invasive germ cell tumours in infertile men. *Histopathology*, **2**, 157.

VAITUKAITIS, J. L., BRAUNSTEIN, G. D. & ROSS, G. T. (1972) A radioimmunoassay which specifically measures human chorionic gonadotropin in the presence of human luteinizing hormone. *Am. J. Obstet. Gynecol.*, **113**, 751.

WALDMANN, T. A. & MCINTIRE, K. R. (1974) The use of a radioimmunoassay for alpha-fetoprotein in the diagnosis of malignancy. *Cancer*, **34**, 1510.

WHITAKER, R. H. (1970) Management of the undescended testis. *Br. J. Hosp. Med.*, **4**, 25.